# 3DPotatoTwin: a paired potato tuber dataset for 3D multi-sensory fusion^☆^

**DOI:** 10.1016/j.plaphe.2025.100123

**Published:** 2025-10-06

**Authors:** Haozhou Wang, Pieter M. Blok, James Burridge, Ting Jiang, Minato Miyauchi, Kyosuke Miyamoto, Kunihiro Tanaka, Wei Guo

**Affiliations:** a Graduate School of Agricultural and Life Sciences, University of Tokyo, 1-1-1, Midori-cho, Nishi-Tokyo, Tokyo, 188-0002, Japan; b Technology Innovation R&D Dept.I, Kubota Corporation, 1-11, Takumi-cho, Sakai-ku, Sakai-shi, Osaka, 590-0908, Japan

**Keywords:** RGB-D camera, Structure from motion, Point cloud registration, Sensor fusion, Dataset

## Abstract

Accurate 3D phenotyping of agricultural produce remains challenging due to the trade-off between reconstruction quality and acquisition throughput in existing sensing technologies. While RGB-D cameras enable high-throughput scanning in operational settings like harvesting conveyors, they produce incomplete, low-quality 3D models. Conversely, close-range Structure-from-Motion (SfM) produces high-quality reconstructions but is not suitable for high-throughput field application. This study bridges this gap through *3DPotatoTwin*, a paired dataset containing 339 tuber samples across three cultivars collected in Hokkaido, Japan. Our dataset uniquely combines: (1) conveyor-acquired RGB-D point clouds, (2) ground measurement, (3) SfM reconstructions under indoor controlled environment, and (4) aligned model pairs with transformation matrices. The multi-sensory alignment employs an semi-supervised pin-guided pipeline incorporating single-pin extraction and referencing, cross-strip matching, and binary-color-enhanced ICP, achieving 0.59 ​± ​0.11 ​mm registration accuracy. Beyond serving as a benchmark for 3D phenotyping algorithms, the dataset enables training of 3D completion networks to reconstruct high-quality 3D models from partial RGB-D point clouds. Meanwhile, the proposed semi-automated annotation pipeline has the potential to accelerate 3D dataset generation for similar studies. The presented methodology demonstrates broader applicability for multi-sensor data fusion across crop phenotyping applications. The dataset and pipeline source code are publicly available at HuggingFace and GitHub, respectively.

## 1 Introduction

Potato (*Solanum tuberosum* L.) is currently one of the most important food crops in the world, after rice and wheat [1]. It has been widely grown for food, seed tuber production, animal feed, and industrial uses. Due to the richness of carbohydrate, energy, Vitamin B complex and C, with little fat in potato tuber, more and more people are choosing it as a staple in the diets. Meanwhile, the increase of world population also puts an urgent need for doubling current yield gains to ensure the food security. Classical breeding of new potato cultivars remains primarily dependent on empirical experience and the selection of simple phenotypic traits [2]. To provide more useful information for validating new varieties, there is an increasing need for precise and efficient modeling of morphological shapes and measurement of advanced morphological traits.

With the development and affordability of sensing techniques, commercial RGB-color cameras has been used to analyze potato tubers based on their produced 2D images. By using computer vision and machine learning segmentation algorithms, Lee et al. [3]; Lee and Shin [4] detected and counted potato tubers in the field for yield monitoring while Si et al. [5] evaluated the tuber shape. Dolata et al. [6] segmented individual potatoes on the conveyor and then used simulation-based learning to obtain physical dimensions and yield monitoring. However, due to the dimensional reduction when projecting a 3D object onto a 2D image, most image-based methods can only evaluate potato tuber shapes using basic 2D morphological traits. Traits such as width, height, and minimum diameter can be extracted from a 2D image using techniques like bounding box or ellipse extraction, although these are not that accurate. Estimating volume or mass, which is difficult to derive directly from 2D images, often involves correlating these simple morphological traits with manually measured data. The accuracy of such volume estimation approaches is frequently questioned.

Advances in 3D scanning and reconstruction technologies have enabled the capture of complex plant structures. While numerous 3D reconstruction methods and sensors are available, cost considerations and technical complexity often guided researchers toward more accessible options. While direct high-precision 3D scanning methods using laser technology certainly exist, many of these solutions come with significant costs. For instance, desktop scanners like the Matter and Form THREE (approximately $1500-$2400) or the Shining 3D EinScan SP V2 (approximately $2500) offer high accuracy. Even more advanced systems like the Artec Micro II can cost around $22,500. Handheld scanners such as the Revopoint POP series (e.g., POP 2 ​at approximately $400) or the Creality CR-Scan Otter (approximately $900) are more affordable but still represent an investment. Additionally, these devices often require a controlled scanning environment and scanning approaches with limited flexibility. As a result, stereo-photogrammetry (Structure-from-Motion/Multi-View Stereo, SfM-MVS) and depth sensors (with the RGB-D cameras as the most prominent) are widely used in many studies due to their lower device cost and ease of operation. SfM methods only require common cameras and photogrammetry processing software, while RGB-D sensors can directly capture images and 3D results.

The SfM photogrammetry approach offers high quality but often low efficiency. It involves extracting and matching feature points from different view images, estimating their relative positions, densifying them into a dense point cloud using multi-view stereo, and converting the point cloud into mesh models with corresponding textures. The measured area ranging from large fields [7] to individual plant [8] and to plant organs [9]. When focusing on organ-level 3D reconstruction in the close-range, to ensure the completion of object scanning and scanning efficiently, often multiple cameras, a rotation table, and a photo studio are often required to capture enough views of objects [10]. The produced 3D model quality is high under the controlled condition, but even with multiple cameras, the data collection and processing speed is slow and cumbersome.

In contrast, depth sensors, particularly when combined with RGB images in RGB-D cameras, offer high efficiency but often low quality. Several low-cost and lightweight commercial products are available, including Intel RealSense (Intel Corporation, California, U.S.), Azure Kinect (Microsoft, Washington, U.S.) and ZED camera (StereoLabs, San Francisco, USA). The primary outputs for these depth sensors are a 2D depth image and/or 2D RGB image. This depth image can subsequently be converted into a 3D point cloud and modeled in 3D by using the pinhole camera model and the camera intrinsics. For potato tuber studies, Long et al. [11] investigated the feasibility of using RGB-D cameras to estimate the volume of potato tubers. Su et al. [12] utilized depth cameras to estimate the thickness and predict the mass of potato tubers, and further extended their research to quality grading using machine vision [13]. Although these studies successfully captured 3D surfaces of potato tubers, the bottom portion of the tubers remained obscured and invisible. Consequently, depth sensor-based approaches could only reconstruct the upper half of the potato. This limitation could introduce potential errors in volume and mass estimation, particularly for irregularly shaped potato tubers [11]. Thus, solving the problem of occlusion that causes incomplete data and improving data quality and efficiency are urgent for the actual application of precision agriculture, not just limited to potato tubers.

To better address the incomplete shape, advanced machine learning algorithms often requires to be developed on a incomplete-complete paired dataset. Traditional methods, such as those relying on symmetry assumptions [14] or super-ellipsoid matching [15], use manually defined rules based on object features. However, these handcrafted approaches are organ-specific, labor-intensive, and struggle with the irregular geometries and high morphological variability of crops like potato tubers. To overcome these challenges, recent work has turned to deep learning methods, which automatically learn and extract shape features from data, thereby reducing reliance on explicit geometric rules. For example, Tang et al. [16] proposed LakeNet for furniture point cloud completion, while Park et al. [17] introduced DeepSDF to infer complete 3D shapes from partial RGB-D inputs. DeepSDF is a 3D shape completion network that has also been used for completing agricultural products: sweet pepper [18], strawberry [19], grape [20], and even potato [21]. Despite the potential, training effective deep learning models demands high-quality and domain-specific datasets. Transferring existing industrial datasets like furniture are not directly applicable to agricultural shape completion tasks. Thus, there is a critical need for specialized paired potato dataset, which combines partial and complete 3D representations of the same potato tubers, to facilitate robust network training and benchmarking.

A proper pairing method across different scans views and sensors is also important, as it can accelerate annotation when preparing paired datasets and contribute to the fusion of results from multiple sensors. For example, Sampaio et al. [22] combined 3D models of in-field individual maize plants, captured with an RGB-D camera, with data on temperature, humidity, and luminosity to provide a more comprehensive understanding of maize growth stages. Such multi-sensor fusion approach can also combine the advantages of SfM and RGB-D sensor, achieving the quality of SfM with the scanning speed of depth sensors. However, integrating data from different sensors presents significant challenges to traditional Iterative Closest Point (ICP) algorithms due to variations in color and shape characteristics, even among sensors of the same type under different lighting conditions or limited overlap. To address this, Isachsen et al. [23] integrated the Absolute Trajectory Error (ATE) into their method, while Guo et al. [24] modified the SAC-IA algorithm with ICP to better align 3D fruit models scanned from different views. In addition to an object's geometry, surrounding objects can also serve as useful references for registration. For example, Zhou et al. [25] employed three white calibration spheres, and Zhang et al. [26] used conical surface fitting of pots assist ICP to align and register multiple point cloud scans in crop studies. Utilizing surface colors and textures offers another registration approach for aligning multiple scans. Yuan et al. [27] used a color-guided ICP algorithm for registering peach tree scans from UAV-mounted LiDAR, while Wan et al. [28] developed a robust LAB color space ICP registration for complex vegetation. Despite the great potential shown by these studies, an available open-source pipeline, feasible for potato tubers and capable of accelerating paired dataset generation, is still missing.

Additionally, actual potato tuber harvesting scenarios present further challenges. First, although RGB-D cameras can capture tuber geometry on conveyor belts at speeds of approximately 0.5–1.2 ​m/s, the resulting scans often contain significant occlusions and noises. Second, during the initial RGB-D scan on the conveyor, tuber surfaces are covered in soil. They are later cleaned to expose the original surface for an accurate ground truth scan. This process alters their visual texture, color, and even shape. Third, marker-based approaches have practical limitations. While multi-point registration theoretically requires over three reference markers, the conveyor's vibration and tuber rotation only permit the reliable placement of a single marker without compromising operational workflow. These challenges increase both the difficulty and value of creating high-quality, well-paired 3D potato tuber datasets from multiple sensors and scenarios.

Thus, we acquired a paired dataset of potato tubers via low quality outdoor RGB-D camera imaging on harvest conveyor belt and high quality indoor close-range stereo photogrammetry (SfM-MVS). The contributions of this study are as follows:

1. **The paired 3D Dataset**: a paired potato tuber 3D point clouds dataset scanned from both RGB-D (incomplete, low quality but high-throughput) and SfM reconstruction (complete, high quality, but low-throughput), supplemented by corresponding ground-truth measurements (e.g. axis length, width, depth, volume, weight), high-resolution RGB images, depth images, and transformation matrices for each tuber pair's spatial relationship;
2. **Semi-automated 3D alignment annotation pipeline**: a semi-automated pipeline with a simple interactive user interface (UI) for aligning and registrating two separate 3D point clouds obtained from RGB-D camera and SfM photogrammetry techniques, despite their differences in shape, texture color, resolution and acquisition method, time and location.

## 2 Methods and materials

This section first introduces the conditions in which the data was acquired in the field and on the harvester (Subsection 2.1). Subsequently, the setups for RGB-D data collection are described (Subsection 2.2). The process for performing manual measurements is then outlined (Subsection 2.3). Next, the devices and methods used for close-range stereo photogrammetry reconstruction (SfM-MVS) are detailed (Subsection 2.4). Finally, after acquiring the 3D data, the semi-automated 3D data alignment annotation pipeline implements a single pin-guided and color-based ICP algorithm is explained, along with the user interface designed for interactive manual operations (Subsection 2.5).

### 2.1 Field condition and harvesting devices

**Fig. 1:**
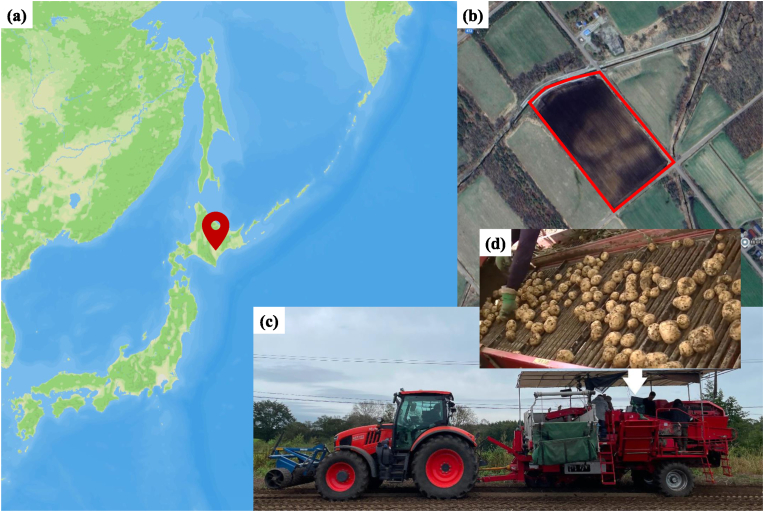
Plot locations and harvesting devices. (a) The potato farmland is located in Sarabetsu Village, Hokkaido, Japan (42.610316N, 143.156753E); Its boundary is shown in (b); (c) The Toyonoki Top-1 single-row potato tuber harvester; and (d) is a view of the conveyor belt.

The potato tubers were grown in a farmland located in Sarabetsu village, Hokkaido prefecture, Japan (Fig. 1a–b), at coordinates 42.610316N latitude and 143.156753E longitude. A Toyonoki Top-1 single-row potato harvester (Fig. 1c), equipped with a conveyor belt (Fig. 1d), was employed for the harvesting process. The potato cultivar Sayaka was grown within the field, the rows were spaced 0.75 ​m apart, and 12 rows were selected randomly for conducting the dataset collection experiment. Those Sayaka tubers were sampled on September 14 and 21, 2023. To increase the diversity of potato sizes and shapes, we also collected data by operating the harvester in the farm's barn. On September 15, we dumped boxes of the Kitahime potato cultivar onto the conveyor belt, and on September 22, we did the same with the Corolle cultivar.

### 2.2 Partial low-quality 3D data collection by a RGB-D camera

**Fig. 2:**
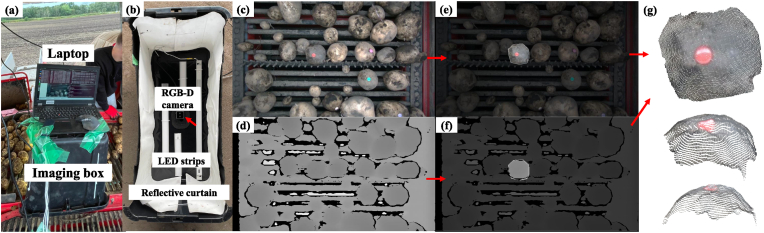
Devices and outputs for RGB-D data collection on conveyor belt. (a) Devices used for data collection on the conveyor belt; (b) The internal setup of the imaging system for providing good lighting condition; (c) An example of an RGB image captured by the RGB-D camera; (d) An example of a depth image captured by the RGB-D camera; (e–f) An example of an annotated RGB and depth image of a single tuber, stored in PNG format in the published dataset, with the mask included as an alpha layer; (g) The extracted 3D partial point cloud from three different views of a potato tuber.

We developed an imaging system for RGB-D data collection on a conveyor belt (Fig. 2a). The system was enclosed within a modified black plastic box with dimensions of 85 ​× ​45 ​× ​39 ​cm (width, depth, and height, respectively). To enhance lighting conditions inside the box, four LED light strips with a color temperature of 6000K were fixed to the interior ceiling (Fig. 2b). A single Intel RealSense D405 RGB-D camera was positioned in the center of the box to capture top-view RGB-D images. The camera was operated using the Robot Operating System 2 (ROS2, Humble Hawksbill version), which was installed on an Ubuntu 22.04 system running on a Lenovo ThinkPad P53 laptop. The RGB-D camera was configured to capture both RGB and depth images at a resolution of 1280 ​× ​720 pixels, 30 frames per second (FPS), and with an exposure time of 5 ​ms to reduce motion blur (Fig. 2d–e). Considering the conveyor belt speed, each potato tubers was captured around 20–30 frames. Initially, the captured images were stored as ROS2 bag files. However, for ease of use in the published dataset, they were converted into separate RGB and depth image files in PNG format.

The region of each target potato tuber (with a reference pin) was annotated with the LabelMe software across all frames (Fig. 2e–f). For each tuber, its mask was assigned a value of 255 in the alpha layer of the PNG file, while the alpha values of the background were set to 0. Lastly, the 3D point cloud of each tuber was generated from the annotated depth images and colored using the corresponding RGB pixels (Fig. 2g). This process was performed using the Python Open3D package and the camera's intrinsic parameters [29], resulting in the final collected RGB-D data. Since only the upper surface is visible, this RGB-D approach collected the partial 3D point cloud data.

**Fig. 3:**
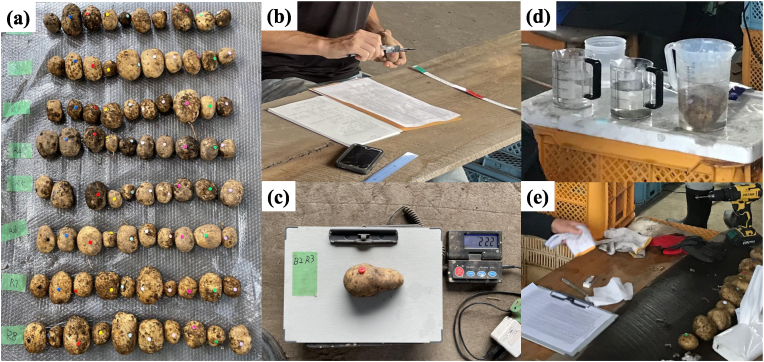
Manual measurements conducted in the barn. (a) all sampled potato tuber of one day harvest; (b) length measure by digital calipers; (c) weight measure; (d) volume measure by water displacement method; (e) surface cleaning and hole drilling.

Despite the ability of the RGB-D imaging system to capture all potato tubers and their 3D point clouds at 30 FPS, it was not practical to perform subsequent manual measurements and SfM-MVS reconstruction for all tubers. Therefore, potato tubers were randomly selected by inserting single colored pins (Figs. 2c and 3a) into them before they passed through the RGB-D imaging system. The pins were round-head thumbtacks, also known as push pins, with an 11 ​mm diameter and an approximate head thickness of 1 ​mm, resulting in a volume of about 0.095 ​ml. This volume increase is negligible compared to the total volume of a potato tuber. After image acquisition, another researcher manually collected these marked tubers and stored them in bags for further indoor processing.

### 2.3 Ground truth measurements

A total of 339 potato tubers of varying sizes and shapes from three cultivars were sampled and marked as the source for the dataset. Ten different pin colors were used as one group, and all tubers sampled on the first day are shown in Fig. 3a. The three-axis dimensions of each tuber, also referred to as length, width, and depth, were measured using digital calipers (Fig. 3b). The tuber weight was recorded (Fig. 3c), followed by tuber volume measurements using the water displacement method (Fig. 3d). Finally, the surface of each potato tuber was wiped with tissue, and a hole was drilled using a handheld drill (Fig. 3e) to allow fixing onto a support for conducting Structure from Motion (SfM) reconstruction.

### 2.4 Complete high-quality 3D data collection by SfM

To obtain a complete and high-resolution 3D model of each potato tuber, we utilized Structure-from-Motion (SfM) reconstruction with the Metashape software. Multiple images of each tuber were captured from various angles under controlled imaging studio conditions. These images were subsequently preprocessed and used to reconstruct a 3D point cloud and generate 3D meshes for the potato tubers in Metashape (Agisoft LLC, St. Petersburg, Russia). All the batch processing scripts mentioned in this section were provided in the *3dscan* folder at Github (https://github.com/UTokyo-FieldPhenomics-Lab/PotatoScan/).

**Fig. 4:**
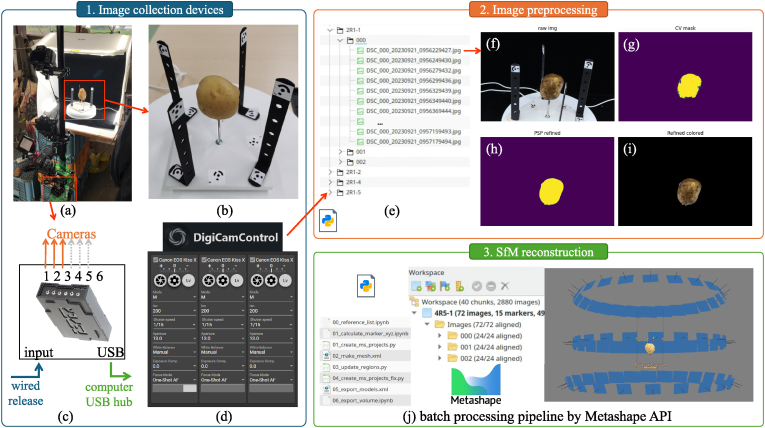
The workflow for semi-automatic 3D reconstruction of potato tuber using stereo photogrammetry (Structure from Motion and Multi-view stereo, SfM-MVS). (a) the commericial imaging studio for multi-view data collection; (b) the marker frames for image alignment; (c) the multi-camera shutter controller and image data transformer; (d) the open-source DigiCamControl software for camera configuration and image renaming; (e) the data structure for image data storage; (f–i) potato tuber segmentation preprocessing; (j) the automatic batch reconstruction pipeline using Metashape software python API.

The imaging studio setup consisted of three Canon X7 DSLR cameras, an automated turntable, four LED lights, and a portable photo studio (Foldio360 by OrangeMonkie Inc.) to ensure a controlled environment (Fig. 4a). To capture the full longitudinal cross-section of each potato tuber effectively, each tuber was mounted on a long threaded screw secured to a white wooden fixed to the turntable (Fig. 4b). To facilitate accurate camera alignment, Metashape's 12-bit circular automatic detectable markers were strategically placed around the setup, with some markers positioned on the narrow threaded bolt at the tuber's hovering height (Fig. 4b). For comprehensive image capture with sufficient overlap, the turntable was controlled by the official app to rotate at 15-degree intervals. At each interval, it briefly stopped for 2 ​s to allow the cameras to capture images. The three DSLR cameras were mounted on tripods and positioned at different heights and viewing angles. They were synchronized using an Esper TriggerBox (Fig. 4c), enabling all cameras to simultaneously take photographs upon pressing a button on the connected trigger controller. The cameras were consistently configured using the open-source software DigiCamControl (https://digicamcontrol.com/). Setting parameters included manual mode, ISO of 200, shutter speed of 1/15, aperture of f/13.0, manual white balance, exposure compensation of 0.0, and single-shot autofocus mode (Fig. 4d). DigiCamControl also renamed the captured images automatically upon transfer to a cached folder, using the naming format: DSC_[camera_id]_[date]_[time][unique_id]. A Python script was later used to organize the cached image files into a designated directory with subfolders categorized by tuber ID and corresponding cameras for ease of later access and analysis (Fig. 4e).

To isolate the potato tuber models and remove the influence of the background, segmentation was performed on the tuber regions in the images. Since the environment was controlled and the background was relatively simple (Fig. 4f), a color-based filtering method was initially used to obtain a rough mask of the tubers. Potato tubers typically exhibit yellow shades, which are best identified in the CIELAB color space. In the *La*∗*b*∗ color space, yellow is characterized by high positive values in the *b*∗ channel and near-zero values in the *a*∗ channel. Setting a threshold of *b*∗ > 15 generated an initial rough mask of the tubers (Fig. 4g). However, challenges such as soil residue and light reflections on the tuber surface sometimes resulted in black or white spots similar to the background. To address this, we directly applied the pretrained CascadePSP [30] deep learning segmentation refinement network to enhance the initial mask. Although the network had not been trained on our dataset, it performed well due to the distinct boundaries of the objects in the images (Fig. 4h–i).

We developed an automated batch processing pipeline using the Metashape Python API for 3D reconstruction (Fig. 4j). Initially, the required tuber images and corresponding generated tuber region masks were loaded into Metashape. One tuber was defined as one chunk in Metashape and each view angles from the same camera were grouped as a camera group. Subsequently, 12-bit circular markers were automatically detected as control points using Metashape's built-in functions. The distances between specific markers were imported as scale bars to ensure accurate scaling of the 3D tuber models. Next, the images were batch-aligned, and corresponding key points were generated. The point densification and mesh generation functions provided by Metashape were then applied to produce 3D point clouds, meshes, and textures of the potato tubers. Finally, minor noise in the 3D models was manually identified and removed using open-source software CloudCompare (https://www.danielgm.net/cc/) to ensure clean and precise representations of the potato tubers. In total, 339 potato tubers were successfully scanned and modeled. All models were processed into watertight meshes using MetaShape's built-in hole-filling function.

We also developed a Python-based pipeline to calculate morphological traits from 3D plant mesh models generated in Agisoft MetaShape. The pipeline processes mesh data and computes the following key traits:

*Three Axis Lengths (cm)*: The major, intermediate, and minor axis lengths, derived from the Open3D axis-aligned bounding box.

*Surface Area (cm*^*2*^*)*: The total external surface area of each plant mesh, calculated using Metashape Python API chunk.model.area().

*Volume (cm*^*3*^*)*: The enclosed volume of each plant mesh, calculated using Metashape Python API chunk.model.volume().

*Volume-to-Surface ratio*: The ratio of potato volume to surface area. Spherical potatoes have the highest ratio, while more irregular shapes have lower ratios.

*Aspect Ratio*: The ratio of the longest to shortest bounding box dimensions (*L*_max_/*L*_min_). This index close to 1 indicates a shape approaching spherical, and the larger the value, the more the shape deviates from spherical.

*Sphericity Index*: Defined as $\sqrt[{3}]{36\pi V^{2}}/A$, where *V* is volume and *A* is surface area. This index ranges from 0 to 1, with 1 representing a perfect sphere.

*Convexity Index*: The ratio between mesh volume and its convex hull volume. Values range from 0 to 1, where 1 indicates a smooth surface, while a smaller value indicates more concave regions (i.e., valleys).

### 2.5 Semi-automated 3D alignment annotation pipeline

The proposed semi-automated annotation pipeline comprises three main steps: 1) Referencing pin segmentation for both RGB-D and SfM point clouds; 2) Stepwise rough matching based on the minimum cross-strip area error around the pin; 3) Fine matching using color-based Iterative Closest Point (ICP) with semi-supervised manual inspection. The python scripts were available in *03_sfm_rgbd_registration* of the *3dscan* folder at Github (https://github.com/UTokyo-FieldPhenomics-Lab/PotatoScan/).

#### 2.5.1 Reference pin segmentation

Since the data sources for RGB-D and SfM differ, we implemented distinct pin segmentation approaches for each method. For the RGB-D method, the partial 3D point cloud data is generated by combining RGB images with depth images from a single view. Transferring a mask from an RGB image to the generated 3D point clouds is straightforward. Therefore, we projected the annotated masks onto the corresponding point clouds. In contrast, the SfM multi-view approach is more complex due to its multi-view nature and the need to handle occlusions. For this method, we directly segmented the 3D point cloud using color information. Specifically, we applied iterative HSV color space thresholding and denoising to ensure accurate segmentation.

**Fig. 5:**
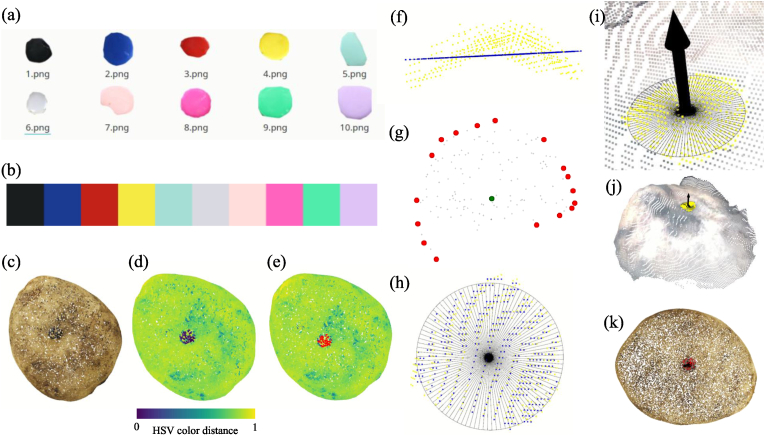
Illustration of the pin segmentation process. (a) Pin reference images used for calculate median HSV values; (b) Median HSV values of pins from reference images; (c) Source potato tuber with black pins in the center, the most difficult case; (d) Calculated HSV color distance; (e) Segmentation results with threshold ​= ​0.25; (f) Projection of pin point cloud onto 2D plane using minimum bounding box; (g) Pin center calculation through convex hull bounding points and hyperLSQ circular fitting; (h) Visualization of pin plane and circular fit; (i) Pin plane with normal vector pointing outward; (j) Results on RGB-D 3D model; (k) Results on SfM 3D model.

The core of the segmentation process involved iteratively adjusting the color distance threshold to isolate the pin region. First, we prepared a set of reference images for all pins, containing only the pin region with the background removed (Fig. 5a). Since the potato tubers were labeled in the same order as the pin colors (Fig. 3a), the reference image file for each tuber's pin can be directly obtained using its ID. Its median HSV value **c**_ref_ = (*h*_ref_, *s*_ref_, *v*_ref_) was then calculated from the reference pin image to represent its color characteristics (Fig. 5b).

For each point **c**_*i*_ = (*h*_*i*_, *s*_*i*_, *v*_*i*_) in the tuber point cloud **C** ​= ​{**c**_1_, **c**_2_, *…*, **c**_*n*_}, the color distance *d*_*i*_ between **c**_*i*_ and **c**_ref_ was computed by:

$$d_{i}=\overset{3}{\underset{j=1}{\sum }}\Delta c_{ij}^{\text{weighted}}$$

where *j* represents different components in HSV color space, and the weighted differences were given by:

$$\Delta c_{i}^{\text{weighted}}=\Delta c_{i}\cdot w=\Delta c_{i}\cdot \left(0.5,0.1,0.3\right)$$

where, Δ**c**_*i*_(Δ*h*_*i*_, Δ*s*_*i*_, Δ*v*_*i*_) ​= ​|**c**_*i*_ ​− ​**c**_ref_| = (|*h*_*i*_ ​− ​*h*_ref_|, |*s*_*i*_ ​− ​*s*_ref_|, |*v*_*i*_ ​− ​*v*_ref_|). The weights (0.5, 0.1, 0.3) were chosen based on the relative importance of each component manually in the pre-experiment.

Since hue (*h*) was a circular component, the difference must account for this periodicity. Specifically, if the absolute difference in hue exceeded 0.5 (half of the range), it should wrap around:

$$\Delta h_{i}=\left\{\begin{array}{ll} 1-\Delta h_{i}\; & \text{if ​}\Delta h_{i}>0.5,\\ \Delta h_{i}\; & \text{otherwise.} \end{array}\right)$$

This color distance *d*_*i*_ was then normalized between 0 and 1, and an initial threshold of 0.35 was applied to select points likely to belong to the pin. We then calculated the convex hull volume of the selected points to check for outlier noise. Noise was removed using radius outlier filtering with a minimum of 40 points and a radius of 5 ​mm. For the denoised points, the convex hull volume was recalculated. If the volume still exceeded the predefined limit (60 ​mm^3^), the threshold was decreased by 0.05, and the process was repeated until the pin region was narrowed without noise. For example, Fig. 5e shows the segmentation result obtained at a threshold of 0.25, where the convex hull volume satisfies the required condition.

After segmenting the pin area, which served as a reference point for aligning two point clouds, it is necessary to compute the circular center and the outward-pointing normal vectors. To calculate the center, we first determined the oriented bounding box of the 3D convex hull derived from the pin point cloud. The perpendicular bisector plane of the shortest axis of this oriented bounding box was used as a reference plane. All 3D points of the pin were then projected onto this plane to obtain a 2D representation (Fig. 5f). Next, the hyperLSQ circle fitting algorithm (https://github.com/AlliedToasters/circle-fit) was applied to find the center of the convex hull points (Fig. 5g). The fitted radius and reference plane were visualized in Fig. 5h). To compute the outward-pointing normal vector, all points were projected along the normal vector axis to identify two endpoints. The endpoint closest to the pin's center was selected, ensuring the normal vector's direction was opposite to the other endpoint (Fig. 5i). The final results of the pin segmentation, including the segmented pin points, pin center, radius, plane, and outward-pointing normal vectors, were shown for RGB-D 3D point clouds and SfM point clouds in Fig. 5j and k, respectively.

#### 2.5.2 Stepwise cross-strip rough matching

**Fig. 6:**
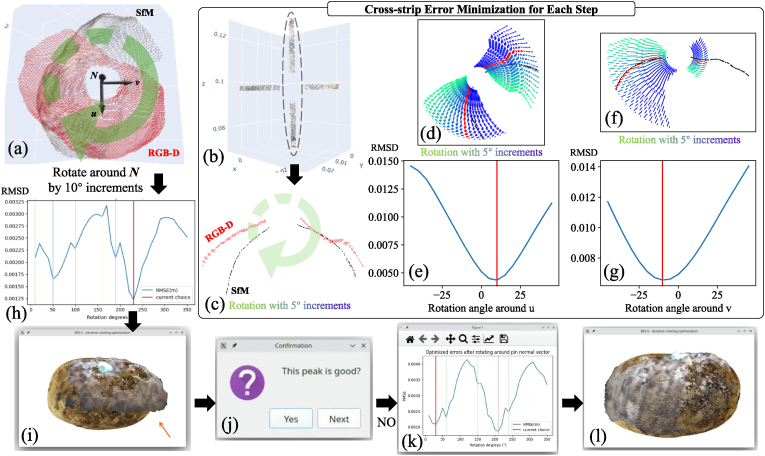
Cross-strip alignment for potential rotation matrix estimation: (a) Rotate around pin normal vectors (*N*) in 10° increments. For each step, apply the cross-strip error minimization to determine the optimal *v* and *u* angles; (b) Extract the cross-strip region around the pin neighborhood; (c) The strip derived from vector *u*; (d) & (f) Rotate the RGB-D point cloud strip in 5° increments; (e) & (g) Compute the RMSD between RGB-D and SfM strips after each rotation step, where red indicates angles with minimum errors; (h) Using the optimal *u* and *v* rotation angles in previous cross-strip error minimization, calculate the total errors after each rotation step around pin normal vectors. The local minimum are chosen as potential rotation angles (colored vertical lines); (i) The global minimum is not always the best choice for rotation angles; (j) An interactive UI is provided for manual inspection; (k) The second-rank local minimum matches the actual results from manual inspection in (l).

Two tuber point clouds were initially aligned using the center position and normal vector of reference pins (Fig. 6a). This initialization fixed the x, y, and z positional degrees of freedom, leaving only the rotation angle undetermined. To achieve better alignment, we adjusted the rotation around the normal vector (*N*) and its associated tangent (*u*) and bitangent (*v*) vectors between the two point clouds (Fig. 6a). To minimize the effects of the tuber's backside and focus only on the area around pins, we used only the neighboring region within a 3 ​cm radius from the pin center for subsequent analysis.

We rotated the RGB-D point cloud around *N* and pin center point by 10° increments. For each stepwise rotation around *N*, we applied the cross-strip error minimization to obtain the optimal rotation angles around *u* and *v*. We first extracted the cross-strip regions with the same *u* and *v* directions (Fig. 6b). The strip widths were set to 1 ​mm in this study. For strips with the same vector direction, we rotated RGB-D ones around pin center point with 5° increments (Fig. 6c). Fig. 6d and 6f shows the stepwise rotation around *u* and *v*, respectively. For each rotation, the root mean square of point distances (RMSD) were calculated to reveal the distance and error between RGB-D and SfM strips. Instead of using the commonly used Chamfer distance, which computes the sum of bidirectional errors [distance(*A* → *B*) ​+ ​distance(*B* → *A*)], RMSD only evaluates unidirectional error [distance(*A* → *B*)], which can also renamed as one-way Chamfer distance. This unidirectional approach is more suitable for our study because we aim to align partial, low-resolution RGB-D data to a complete, high-resolution SfM reference. Furthermore, it reduces computational overhead by half compared to Chamfer distance.

$$\text{RMSD}=\sqrt{\frac{1}{n}\overset{n}{\underset{i=1}{\sum }}\|{\text{source}}_{i}-{\text{target}}_{i}{\|}^{2}}$$

where *n* is the number of points of RGB-D strips, and ‖ ⋅‖ denotes Euclidean distance of two points. The source was one point in RGB-D strips, while the target was the closest point in SfM strips. The rotation angles around *u* and *v* that yield the minimum RMSD values (Fig. 6 e&g) were selected as the optimal angles for each step.

After determining the optimal rotation angles around axes *u* and *v* for a given *N* rotation angle, we computed the RMSD in a 3 ​cm radius region surrounding the pin. By repeating this process for each incremental rotation of *N*, we examined how the RMSD changes with rotation around *N* (Fig. 6h). The optimal rotation angles correspond to local minima (shown by colored vertical lines in Fig. 6h), although the global minimum does not always yield the correct match. Therefore, we incorporated manual verification through an interactive UI to select the appropriate rotation angles (Fig. 6 i&j). In the example shown, the second local minimum (Fig. 6k) provided the best match based on manual inspection (Fig. 6l).

#### 2.5.3 Matching refinement by interactive color-based ICP

**Fig. 7:**
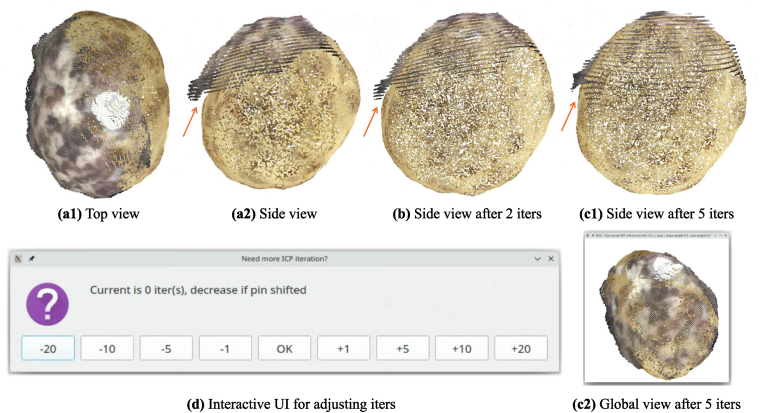
Alignment refinement using interactive color-based ICP iterations. (a1)-(a2) Initial rough alignment without color-based ICP; (b) Result after 2 color-based ICP iterations; (c1)-(c2) Final alignment after 5 color-based ICP iterations; (d) Interactive UI for adjusting iterations and inspecting results (c2).

The initial alignment using pin-guided stepwise matching provided a rough registration for potato tuber point clouds from two scanning methods (Fig. 7a1). However, small gaps often remain between the two point clouds (Fig. 7a2). To refine these minor discrepancies, we employed a color-based ICP algorithm to strengthen the guidance of pin positions. The tuber point cloud was labeled with two colors. We used pure red (255, 0, 0) for pins and pure blue (0, 0, 255) for the tuber surface. Then, both tuber point clouds were down sampled by 1 ​mm voxels to remove the effects of different model resolution. The geometry weight was set to 10 ​% while color weight was 90 ​% to prioritize label consistency. The convergence threshold was set to 0.5 ​mm to terminate iterations when mean correspondence distance falls below this value.

For the iteration setting, different potato tubers have different shapes, their gap size also varies. In our pre-experiment, it was challenging to automatically define the optimal number of iterations. Thus, we implemented the color-based ICP algorithm with a manual interactive iteration setting, users can adjust the number of iterations interactively and inspect the results in real-time to find the best iteration number (Fig. 7b-d). Finally, the transform matrix obtained from color-based ICP was saved to JSON file for further processing.

## 3 Results and discussion

### 3.1 Paired 3D dataset structure

**Fig. 8:**
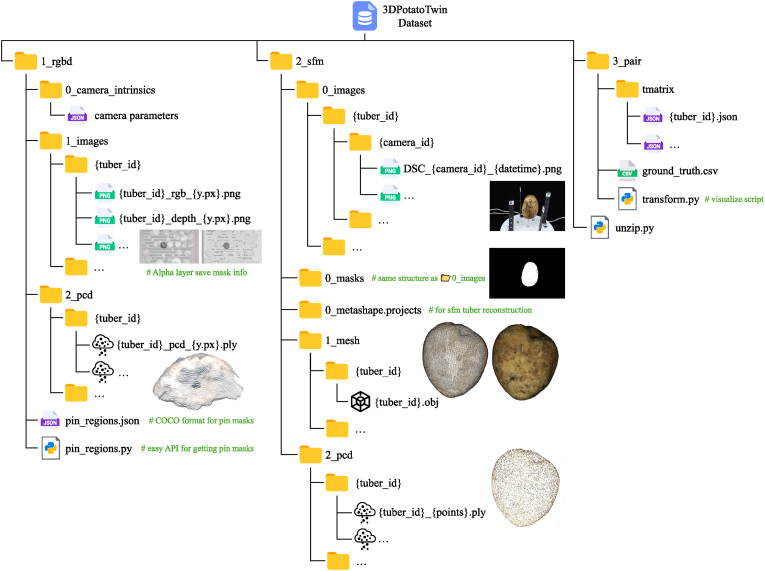
Folder structure of 3DPotatoTwin, containing 3 subfolders. One for RGB-D source data and models, one for SfM source data and models, and one for paired matrix and ground truth data.

We provide all ground measurement data, source image data from both RGB-D and SfM pipelines, and the resulting transformation matrices as a dataset named 3DPotatoTwin on the Hugging Face platform (https://huggingface.co/datasets/UTokyo-FieldPhenomics-Lab/3DPotatoTwin). It enables researchers to develop and test their 3D algorithms and networks. The data are organized into three subfolders: RGB-D source data and models, SfM source data and models, and paired transformation matrices with ground truth data (Fig. 8).

Within the **1_rgbd** directory, the *0_camera_intrinsics* subfolder contains two JSON files storing camera intrinsic parameters. These parameters are essential for accurate reconstruction and mapping in RGB-D image analysis. The *1_images* subfolder stores RGB and depth images organized by {tuber_id}. The files follow a systematic naming convention: {tuber_id}_{type}_{pos} for RGB data and its corresponding depth data. For each potato tuber, all frames captured by the RGB-D cameras were stored. The frames were renamed based on the pixel position of the tuber center along the image's y-axis, with the value 0 being the bottom of the image and 720 being the top of the image. This was renaming was chosen such that the images names were having a sequential order in correspondence with the movement of the potato tubers on the conveyor belt during image acquisition. The image alpha layer provides mask information for individual tuber regions. These segmentation masks are also stored in COCO dataset format in a root folder file named pin_regions.json. A Python script, pin_regions.py, is provided for easy access to these masks. The *2_pcd* subfolder contains 3D point cloud data of the scanned potato tubers. The point cloud files are organized by tuber index and named as {tuber_id}_pcd_{y_pixels}.ply.

The **2_sfm** directory contains source images, project files, and 3D models generated using Metashape software. The 0_images stores the input RGB images for 3D reconstruction, organized by {tuber_id} and {camera_id}. Images were captured using three cameras labeled 000, 001, and 002. The *0_masks* subfolder contains segmentation masks following the same folder structure as *0_images*. Metashape project files for 3D reconstruction are stored in *0_metashape.projects*, with each project containing approximately 50 potato tubers to ensure stable batch processing. The *1_mesh* holds the final textured 3D mesh models exported from Metashape in OBJ format. From these high-quality meshes, we extracted 3D point clouds of individual tubers. We generated point clouds at three densities: 10,000, 20,000, and 30,000 points per tuber. These point clouds are named {tuber_id}_{num_points}.ply and stored in the *2_pcd* subfolder.

In the **3_pair** section, the transformation matrix for aligning RGB-D and SfM tuber point clouds is stored in the tmatrix JSON files. Intermediate data, including parameters from the manual alignment annotation by pin-guided algorithms, the RMSE and our modified one-way chamfer distance (RMSD) between two point clouds are also recorded. A Python script named transforms.py verifies the transformations and evaluates performance. The ground truth measurements are provided in ground_truth.csv, with some sample data shown in Table 1.

**Table 1:** Short summary of potato ground measurements including volumes.

Label	Cultivar	Color	Weight (g)	Length (mm)	Width (mm)	Depth (mm)	CircX1 (cm)	CircX2 (cm)	Volume (ml)
R1-1	Sayaka	black	84	63	50.2	43.2	–	–	80
R1-2	Sayaka	blue	234	104.1	69.1	53.3	–	–	230
R1-3	Sayaka	red	154	73.4	63.1	54.4	–	–	150
⋮	⋮	⋮	⋮	⋮	⋮	⋮	⋮	⋮	⋮
2R1-1	Kitahime	black	346	95	88	55	28	25.6	320
2R1-2	Kitahime	blue	248	80	71	70	25.5	23	230
2R1-3	Kitahime	red	118	58	60	52	19	18.5	105
⋮	⋮	⋮	⋮	⋮	⋮	⋮	⋮	⋮	⋮
5R3-8	Corolle	magenta	100	86	47	37	21	15	80
5R3-9	Corolle	green	188	98	61	56	25.5	18.5	150
5R3-10	Corolle	purple	136	80	55	46	21	17	120

Note: CircX1 represents major axis circumference; CircX2 represents minor axis circumference. Missing values are marked with –.

### 3.2 Dataset variations

**Fig. 9:**
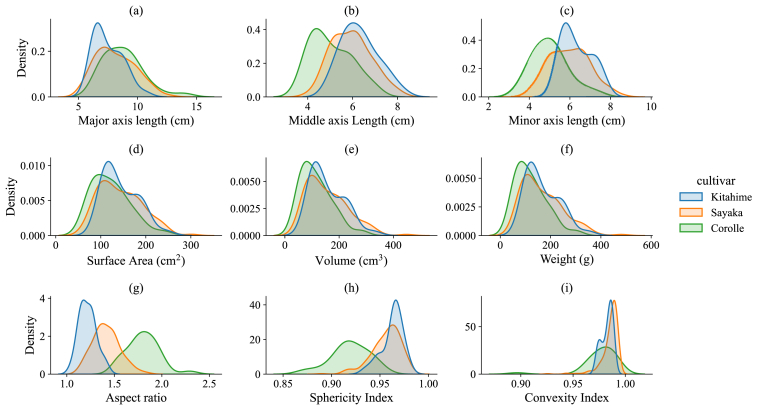
Kernel density estimates (KDEs) for visualizing the traits distribution of potato tuber dataset. Please note, these KDEs are computed independently for each cultivar. Density values are not directly comparable across features due to differences in data scales (e.g., length vs. convexity index).

Fig. 9 shows the distribution of several morphological traits to illustrate the variability in the potato tuber dataset. The length variations of tuber axes are presented in Fig. 9a–c. The longest tuber length ranges from 4.92 ​cm to 13.90 ​cm (Fig. 9a), with volumes ranging from 33.97 ​cm^3^ ​(ml) to 457.89 ​cm^3^ ​(ml) (Fig. 9e) and weights from 36 ​g to 494 ​g (Fig. 9f). Potato volume and weight show high correlation, as most cultivars have a density ranging from 1.07 to 1.1 (Abbasi et al., 2019, Table 1a). This trend is also observed on the Japanese sweet potato ([32], Fig. 9). This range covers most common market sizes.

Different potato cultivars exhibit distinct shapes. Kitahime shows greater middle axis length (Fig. 9b) and shorter axis length (Fig. 9c) compared to Sayaka and Corolle. This results in a lower aspect ratio (where 1 represents a perfect sphere, Fig. 9g) and higher sphericity index (Fig. 9h). According to Abbasi et al. [31], the sphericity index ranges from 79.8 ​% (Cardinal) to 94.3 ​% (Lady Rosetta). Corolle exhibits the least spherical shape and shows significant variation in surface smoothness (Fig. 9i).

### 3.3 Precision analysis

**Fig. 10:**
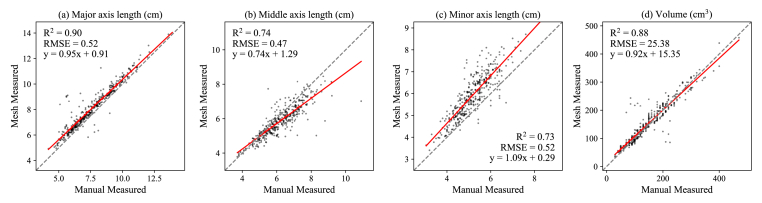
Comparison between manual measured morphological traits and SfM produced Mesh traits.

To evaluate the accuracy of 3D modeling, we compared manual measurements with four overlapping morphological traits derived from 3D SfM models. These traits included lengths along three axes and total volume. (Fig. 10). For the longest axis length and volumes, the manual measurement and 3D approach showed high correlations with R2 around 0.88 and RMSE with 5.2 ​mm and 25.3 ​cm^3^ (Fig. 10a&d). The results for middle axis length and minor axis length exhibited less consistency compared to major axis length. It is primarily because identifying a potato's longest dimension is relatively straightforward, making it easier to measure consistently in both 3D model calculations and manual measurements. However, determining the shortest and intermediate axes proved more challenging in practice. Manual measurements are often less precise due to imperfect orthogonal alignment of the axes, whereas computational bounding box (bbox) calculations assume ideal geometric orientations. Additionally, the bbox-derived minor axis length does not strictly correspond to the physically measured shortest dimension obtained using digital calipers. The bbox method tends to overestimate the thinnest section, which aligns with the overestimation observed in (Fig. 10c). Similar studies support the high accuracy of 3D reconstruction methods. For instance, Liu et al. [33] reported that structure-from-motion (SfM)-based volume estimation achieved an RMSE of 10.4 ​cm^3^, while Huynh et al. [32] demonstrated 97 ​% accuracy in sweet potato measurements using a 3D reconstruction approach. In practical applications, the 3D reconstruction method provides more reliable ground truth than manual measurements. During this study, manual measurements introduced uncontrollable human errors, resulting in several extreme outliers in the data.

Our pin-guided point cloud alignment method, assisted by manual interactive refinement, achieved a modified one-way Chamfer distance (RMSD) of 1.04 ​± ​0.29 ​mm and yielded a RMSE of 0.59 ​± ​0.11 ​mm, calculated by Open3d. In comparison, similar studies on multi-sensor data fusion report varying degrees of accuracy. Wan et al. [28] demonstrated that incorporating color information (via *L*∗*a*∗*b*∗ space and Cauchy kernel) improved ICP performance by three orders of magnitude, reducing alignment error from 45 ​mm (classical ICP) to 2.08 ​mm. Zhang et al. [26] achieved a mean registration error of 1.98 ​mm for maize plants using conical surface fitting-enhanced ICP. Yuan et al. [27] reported RMSE values of 0.05–0.2 ​m for GNSS-IMU-assisted colored ICP in UAV-LiDAR peach tree registration. Xie et al. [34] fused spectral and RGB-D data, achieving an RMSE of 0.4 ​mm. Huang et al. [35] noted a mean error of 2.2 ​mm (maximum 3.3 ​mm) in their fusion framework. While the accuracy of our method is slightly lower than some algorithmic benchmarks, it remains competitive with state-of-the-art fusion approaches, particularly in complex practical application scenarios.

### 3.4 Limitations and future works

While the pin-guided approach demonstrates promising matching results, several limitations remain. The method performs better on irregularly shaped objects compared to more spherical produce, as spherical geometries provide fewer distinctive features for determining optimal rotation angles. Particularly challenging are smooth-surfaced crops like tomatoes and grapes, where both the shape regularity and surface reflectivity complicate 3D data acquisition. A potential solution may involve employing multiple pins for enhanced positioning accuracy, though this would increase preprocessing complexity and pin occlusion. Future work will focus on expanding multi-scale mixed datasets, such as our ongoing broccoli collections combining low-resolution outdoor drone scans with indoor high-resolution reconstructions. Methodological improvements may incorporate deep learning and active learning [36] to reduce manual intervention, along with training set augmentation strategies similar to automatic synthetic weed dataset generation [37].

While RGB-D cameras provide scalability and real-time performance, their inherent limitations, such as partial observations, depth noise, and low resolution, have traditionally limited their use in applications requiring precise 3D reconstruction. Our paired dataset and completion framework show how data-driven approaches can address this accuracy gap: by using high-fidelity SfM references as training targets, networks can learn to compensate for sensor-level inaccuracies while maintaining the speed advantages of RGB-D systems. Building on this dataset, we extended the 3D fruit completion work [17] and proposed a 3D potato tuber shape completion network for partial RGB-D inputs. Our method achieves an average completion accuracy of 2.8 ​mm with 10 ​ms processing time per tuber, showing better volumetric estimation (RMSE: 22.6 ​ml) compared to linear regression (31.1 ​ml) and baseline models (36.9 ​ml) [21]. These limitations apply not only to agricultural applications but also to industrial inspection, logistics automation, and infrastructure monitoring. By integrating high-quality reconstructed data with low-quality sensor data, the performance of robotic or industrial applications can be improved. For example, training on partial scans from Time-of-Flight sensors and RGB cameras, along with CAD models using Monte Carlo-optimized ICP 3D matching, significantly enhances industrial defect inspection [38]. Similarly, Kawka et al. [39] combined partial RGB-D scans with complete LiDAR-based 3D scanning, achieving sub-centimeter precision in pipe diameter inspection for matte and non-reflective surfaces. Additionally, integrating deep reinforcement learning in a digital twin environment, reconstructed via 3D scanning and simulated in visual components, improves camera pose estimation and collision detection [40]. Future work could explore domain adaptation techniques to generalize this approach across different environments (e.g., from controlled agricultural settings to unstructured construction sites) or hybrid architectures that combine RGB-D inputs with inertial or sparse LiDAR data for robustness. Adding 3D point cloud from handheld laser scanner or table laser scanner platform can also expand the data source of multi-sensory fusion. Such advancements would further expand 3D perception capabilities for resource-constrained applications.

## 4 Conclusion

This data article releases a paired 3D dataset and its semi-automated annotation pipeline for pairing potato tuber reconstructions from stereo photogrammetry (SfM-MVS) and RGB-D sensing modalities. Our primary contribution, the *3DPotatoTwin* dataset, comprises 339 tuber samples across three cultivars from Hokkaido, Japan, featuring complete ground truth measurements, source imagery, reconstruction metadata, and aligned 3D models from both sensing approaches. The proposed dataset serves dual purposes as both training data for 3D completion networks and as a benchmark for agricultural vision systems. Our secondary contribution is a semi-automated annotation pipeline that reduces manual annotating effort when aligning 3D models with varying shapes and textures captured by different sensors. This pipeline achieved a fusion accuracy of 0.59 ​± ​0.11 ​mm RMSE and 1.04 ​± ​0.29 ​mm modified one-way Chamfer distance, demonstrating effective alignment despite significant variations in shape representation, texture color, and spatial resolution of potato tuber 3D models. The proposed dataset and annotation pipeline also show great potential for multi-sensor fusion applications across various crop phenotyping scenarios or other applications which employ RGB-D cameras and 3D complete models.

## Authorship contribution statement

**Figure:**
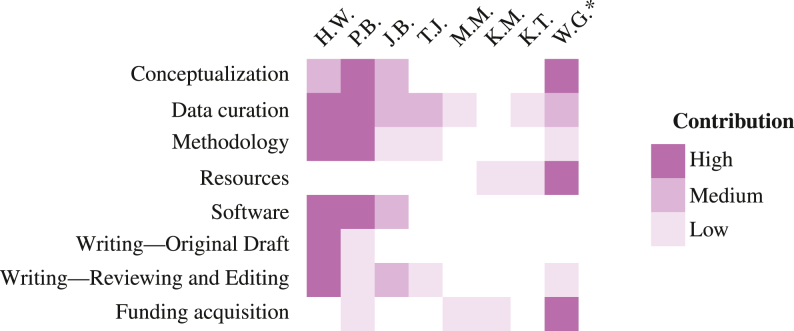

## Declaration of generative AI and AI-assisted technologies in the writing process

During the preparation of this work the authors used ChatGPT and DeepSeek in order to check grammar, spelling, and improve fluency. After using these tool/service, the authors reviewed and edited the content as needed and take full responsibility for the content of the published article.

## Data availability

The dataset and pipeline source code are publicly available at:

• https://huggingface.co/datasets/UTokyo-FieldPhenomics-Lab/3DPotatoTwin
• https://github.com/UTokyo-FieldPhenomics-Lab/PotatoScan/

## Declaration of competing interest

The authors declare that they have no known competing financial interests or personal relationships that could have appeared to influence the work reported in this paper.
